# CAPE Derivatives as Potent Agents for Induction of Osteogenic Differentiation in DPSCs and Biomaterial Development

**DOI:** 10.3390/biomedicines13123039

**Published:** 2025-12-10

**Authors:** Marwa Balaha, Barbara De Filippis, Monica Rapino, Paulina Kazimierczak, Agata Przekora, Tamer Esmail, Eleonora Chiara Toto, Giulia Petrucci, Cristina Canal, Amelia Cataldi, Viviana di Giacomo

**Affiliations:** 1 Department of Pharmacy, University “G. d’Annunzio”, Chieti-Pescara, 66100 Chieti, Italy; eleonora.toto@unich.it (E.C.T.); giulia.petrucci001@phd.unich.it (G.P.); amelia.cataldi@unich.it (A.C.); viviana.digiacomo@unich.it (V.d.G.); 2 Department of Pharmaceutical Chemistry, Faculty of Pharmacy, Kafrelsheikh University, Kafrelsheikh 33511, Egypt; 3 Genetic Molecular Institute of CNR, Unit of Chieti, University “G. d’Annunzio”, Chieti-Pescara, 66100 Chieti, Italy; monica.rapino@unich.it; 4 Department of Tissue Engineering and Regenerative Medicine, Medical University of Lublin, 20-059 Lublin, Poland; paulina.kazimierczak@umlub.pl (P.K.); agata.przekora-kusmierz@umlub.pl (A.P.); 5 Department of Medicine and Aging Sciences, University “G. d’Annunzio”, Chieti-Pescara, 66100 Chieti, Italy; tamershiblali.esmail@phd.unich.it; 6 Department of Materials Science and Engineering, Research Centre for Biomedical Engineering, Universitat Politècnica de Catalunya—BarcelonaTech (UPC), 08034 Barcelona, Spain; cristina.canal@upc.edu; 7 UdA-TechLab, Research Center, University “G. d’Annunzio”, Chieti-Pescara, 66100 Chieti, Italy

**Keywords:** bone regeneration, CAPE, DPSC, biomaterial, stability, natural compounds, synthetic derivatives

## Abstract

**Objectives**: Bone defects, resulting from many causes, represent a challenge in maxillofacial and orthopedic surgery. Regenerative medicine offers promising strategies by introducing exogenous materials to modify the tissue environment and modulate the body’s natural healing mechanisms. Dental pulp stem cells (DPSCs) are considered an effective source for tissue repair. Small molecules such as caffeic acid phenethyl ester (CAPE), although having promising effects in promoting bone regeneration, are characterized by low chemical stability, which impairs their clinical application. This study aimed to investigate the bone regenerative capability of four CAPE derivatives, recently synthesized in our laboratory and selected based on previous studies. **Methods**: DPSCs were induced to osteogenic differentiation in the presence of these compounds (0–5 μM), and cell viability, matrix deposition, alkaline phosphatase activity, and osteogenic marker gene expression were evaluated. In addition, bone biomaterials composed of a chitosan/agarose matrix reinforced with nanohydroxyapatite and enriched with these CAPE derivatives were fabricated and assessed for cytotoxicity and cell adhesion. **Results**: Two of the tested compounds effectively enhanced DPSC differentiation toward the osteogenic lineage. The fabricated bone biomaterials showed no cytotoxicity and supported cell adhesion. Furthermore, these compounds demonstrated stability under various conditions, confirming their suitability for incorporation into bone biomaterials. **Conclusions**: The tested CAPE derivatives exhibit promising osteoinductive properties and stability, offering a valid alternative to traditional therapeutic strategies in regenerative medicine.

## 1. Introduction

Trauma, infections, tumours, biochemical and congenital disorders or abnormal skeletal development can produce bone defects that are the major causes of functional disability [1,2,3]. Unlike most tissues, bone possesses remarkable regenerative abilities, being able to often heal fractures without scarring [4]. The physiological process of bone regeneration involves a finely tuned interaction among cells, the extracellular matrix and growth factors, starting with an inflammatory response and culminating in mature bone formation [5]. However, extensive bone loss or large defects present challenges in orthopedics and maxillofacial surgery as natural healing mechanisms are insufficient, necessitating bone grafts to aid in fracture regeneration [4,6]. Bone healing is promoted by bone grafts, which represent the second most transplanted tissue after blood and induce, alone or in combination with other materials, osteogenesis, osteoinduction, and/or osteoconduction [2]. The selection of an appropriate bone graft depends on various factors such as tissue viability, defect characteristics, biomechanical properties, cost, and potential complications [7]. All natural bone grafts, including autografts, allografts, and xenografts, have either advantages or disadvantages [7,8]. Consequently, alternative options like synthetic and biologically derived tissue-engineered biomaterials have been explored [1,3]. Initially seen as mere physical support for cells, biomaterials were later found to have complex biological functions that promote tissue regeneration by stimulating the body’s natural repair mechanisms and delivering bioactive substances to encourage cell growth and differentiation [9,10,11,12,13].

Regenerative medicine (RM) is a rapidly growing field of medicine that aims to replace, heal, or regenerate human cells, tissues, or organs that have been lost or damaged due to age, disease, or congenital defects [14,15]. RM seeks to accelerate the body’s natural healing process by introducing exogenous materials and biological factors that can modify the tissue environment [16]. Recent efforts have focused on finding small molecule drugs to enhance tissue regeneration and repair by modulating the regenerative process at the molecular, cellular, and tissue levels [17,18].

Stem/progenitor cells are unspecialized cells that are characterized by the ability to self-renovate and to differentiate into different lineages and specific specialized cell types [19]. Stem cells can be found in various human adult tissues and organs, including adipose tissue, skin, hair follicles, bone marrow, blood, and tooth [19]. In particular, dental cells offer a rapidly accessible and effective source of stem cells compared to other tissues like bone marrow [20,21]; they were recognized to differentiate into diverse tissue types both in vitro and in vivo, including bone, odontogenic, endothelial, adipose, and neural-like tissues [19,22,23,24]. Dental pulp stem cells (DPSCs), particularly, possess remarkable regenerative potential, demonstrating the ability to repair diverse tissues including skeletal muscle, neuronal tissue, skin lesions, blood vessels, ischemic tissue, liver, periodontal tissue and bone through their differentiation into various cell types [20,25,26,27,28]. Moreover, DPSCs could be used with artificial bone to improve bone formation for dental implants [29]. Interestingly, these cells exhibit a low expression of Class II HLA-DR molecules, enabling transplantation between individuals without tissue matching [30], thereby suggesting that their clinical potential is not confined to the oral cavity.

**Figure 1:**
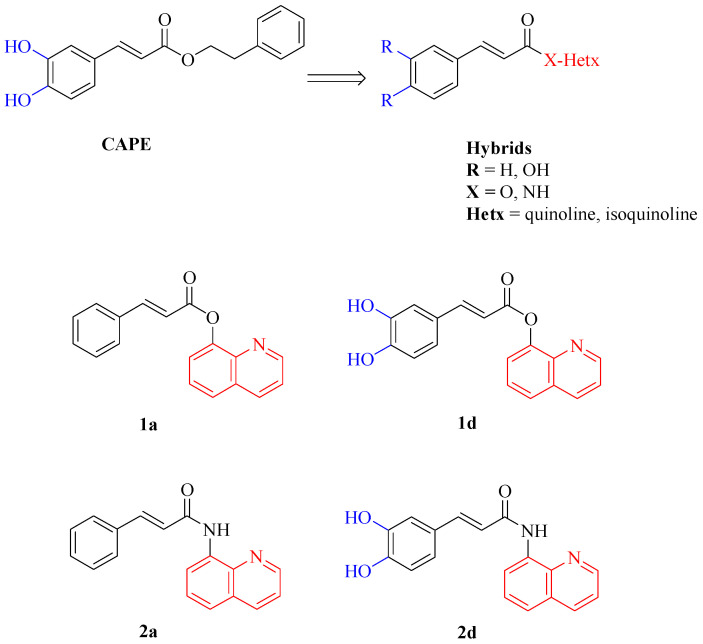
CAPE, hybrid derivatives and compounds **1a**, **1b**, **2a**, **2d** used in the present study.

The polyphenolic caffeic acid phenethyl ester (CAPE, Figure 1), contained in propolis from honeybee hives, possesses diverse biological activities including anti-inflammatory, immunomodulatory [31], antiviral [32], anti-cancer [33], antioxidant and neuroprotective properties [34]. CAPE has shown promising effects on new bone formation and healing in many animal studies, which underscore the potential therapeutic value of CAPE in the field of bone regeneration and repair [35,36,37,38]. Studies have demonstrated CAPE’s ability to prevent bone loss and promote bone repair by acting on both osteoclasts and osteoblasts. Specifically, CAPE has been reported to inverse the decrease in osteoblastic differentiation markers induced by oxidative stress, including Alkaline Phosphatase (ALP) activity, collagen type I release, nuclear phosphorylation of Runx2 and Colony-Forming Unit-Osteoprogenitor (CFU-O) formation [39]. Additionally, CAPE exhibits a dual effect on osteoclasts by suppressing RANKL-induced NF-kB and NFAT activation, where low concentrations inhibit osteoclastogenesis whereas higher concentrations induce apoptosis and disrupt the microtubule network in osteoclast-like cells [39,40,41].

Despite its promising therapeutic properties, the poor stability of CAPE poses a significant challenge for its clinical application, as it is susceptible to hydrolysis both in vivo and in vitro, which limits its efficacy and bioavailability. In addition, temperature and pH notably impact CAPE’s stability, with higher temperatures and/or pH accelerating its degradation [42,43]. Recent research from our lab has focused on synthesizing novel CAPE derivatives with enhanced stability and biological properties [44]. They contain the cinnamic or caffeic acid moiety (blue region in Figure 1) combined by an ester or amide linker to a quinoline or isoquinoline, introduced instead of the benzyl moiety of CAPE (red region in Figure 1). These compounds could be considered hybrid derivatives of CAPE with improved wound healing capacity, highlighting their potential application in regenerative medicine.

Regarding the treatment of bone lesions and wounds, there is a long history of the use of autogenous, allogenic, and xenogenous materials. Therefore, novel reliable materials for bone repair and chronic wound healing are needed. Moreover, regenerative technologies developed in one therapeutic field may be applicable to others, because there is often a consistent similarity in cellular pathways [45]. Based on these data, we aimed to expand our knowledge about the regenerative potential of these new molecules also in the bone regenerative field. To this end, the compounds with the best wound healing effects and chemical and thermal stability [44], i.e., compounds **1a** and **1d** (Figure 1), were selected whereas their amide isosteres **2a** and **2d** (Figure 1) were included in the study to compare the effect of the linker on the biological activities.

Their effect on the osteogenic differentiation of DPSCs was investigated. Furthermore, bone biomaterials enriched with the most promising compounds (**1a** and **1d**) were fabricated. Our goal was to propose innovative strategies for the management of bone defects, exploiting the regenerative properties of these CAPE derivatives.

## 2. Materials and Methods

### 2.1. Cell Culture

#### 2.1.1. Culture of Dental Pulp Stem Cells

Dental pulp stem cells (DPSCs) were cultured in α-MEM medium (Merck, Darmstadt, Germany), supplemented with 10% fetal bovine serum (FBS) and 1% penicillin/streptomycin (Euroclone S.p.A., Milan, Italy). The cells were purchased from Lonza (Lonza Group Ltd., Basel, Switzerland) and kept at 37 °C and 5% CO_2_.

#### 2.1.2. Human Fetal Osteoblast Cell Line

Normal human fetal osteoblast cell line (hFOB 1.19, ATCC CRL-11372) was cultured in a 1:1 mixture of Dulbecco’s Modified Eagle’s Medium and Ham’s F12 Medium, supplemented with 2.5 mM L-glutamine, 0.3 mg/mL G418 (Sigma-Aldrich Chemicals, Warsaw, Poland), and 10% FBS (Pan-Biotech GmbH, Aidenbach, Bavaria, Germany) and maintained at 34 °C (5% CO_2_ in air atmosphere).

### 2.2. Crystal Violet Assay

Cell viability (relative numbers compared to control cells) was determined by a crystal violet assay. After reaching the growth exponential phase, DPSCs were seeded into 48-well culture plates at a density of 8 × 10^3^ cells per well. After 24 h, different concentrations (ranging from 0 to 5 µM) of each compound (**1a**, **1d**, **2a** and **2d**) were added to DPSCs in an osteogenic medium, whereas cells cultured in the same osteogenic medium without CAPE derivatives served as control. After 3, 7, 14, 21, and 28 days, the medium was removed from each well, a wash with PBS was performed and cell were then fixed with glutaraldehyde (1%), and stained at room temperature with a crystal violet solution (0.02%) (both from Thermo Fisher Scientific, Waltham, MA, USA). After 30 min, the cells were rinsed with tap water, and bound crystal violet was dissolved in 70% ethanol. Then, absorbance was read at 600 nm with a spectrophotometer (Multiskan GO, Thermo Fisher Scientific, Waltham, MA, USA). The optical density readings were used to indicate cell survival relative to untreated cultures.

### 2.3. Osteogenic Differentiation

DPSCs were seeded at a density of 3.5 × 10^4^ in 12-well plates in the presence of differentiation medium, which consists of complete α-MEM supplemented with 10 nM dexamethasone, 10 mM β-glycerophosphate and 0.2 mM ascorbic acid phosphate (all purchased from Sigma-Aldrich, St. Louis, MO, USA). At the established experimental times (3, 7, 14, 21 and 28 days), cells and/or supernatants were collected for further analyses.

#### 2.3.1. Alizarin Red S (ARS) Staining

Alizarin red staining was performed after 28 days, by washing twice with PBS with Ca^2+^/Mg^2+^ each well, and fixing the cell monolayer of DPSCs in paraformaldehyde 4% for 15 min at room temperature. After washing twice with deionized water, 40 mM Alizarin Red S solution (Sigma-Aldrich) was added to each well and left at room temperature for 20 min on a shaker, followed by several rinses with deionized water in order to eliminate dye excess. For the spectrophotometric evaluation of calcium level deposits, 800 µL/well of 10% acetic acid was added to each well and then incubated for 30 min in agitation. Wells were then scraped and the sample containing calcium deposits was collected and vortexed. After the addition of hot mineral oil (Sigma-Aldrich), the samples were incubated on ice for 5 min and then centrifuged at 20,000× *g* for 15 min. After the removal of the supernatant, 10% ammonium hydroxide was added, and the optical density of 150 µL aliquots of this solution was measured at 405 nm through a microplate reader (Multiskan GO, Thermo Fisher Scientific).

#### 2.3.2. Alkaline Phosphatase (ALP) Activity

A colorimetric Alkaline Phosphate Assay Kit (Abcam, Cambridge, UK) was used following manufacturer’s instructions. Briefly, 50 µL of 5 mM *p*-nitrophenyl phosphate (pNPP) was added to 80 µL of each supernatant and then incubated for 1 h at room temperature in the dark. After the addition of 20 µL of stopping solution, the absorbance was read at 405 nm wavelength through a microplate reader (Multiskan GO, Thermo Fisher Scientific). The ALP activity (mU/mL/min) was calculated in accordance with the manufacturer’s protocol.

#### 2.3.3. ELISA Analysis of Collagen Type I

The release of collagen type I in cell supernatants was measured by a Human Collagen Type 1 ELISA kit (Cosmo Bio Co., Ltd., Tokyo, Japan; cat. no. ACE-EC1-E105-EX). The absorbance was measured at 450 nm spectrophotometrically (Multiskan GO, Thermo Fisher Scientific). The concentration of collagen type I (µg/mL) was calculated using a standard curve generated with specific standards provided by the manufacturers and normalized with the crystal violet values.

### 2.4. Real-Time RT-PCR

#### 2.4.1. RNA Extraction

After 7, 14, and 21 days of treatment, DPSCs were collected and RNA was extracted by PureLink^®^ RNA Mini Kit (Life Technologies, Carlsbad, CA, USA). An amount of 300 µL of lysis buffer supplied with 1% of 2-mercaptoethanol and then 300 µL of 70% *v*/*v* ethanol were added to each cell pellet, and samples were transferred into the Spin Cartridge for RNA extraction and purification. After washing with Wash Buffer supplied by the kit, samples were incubated for 15 min with 80 µL of DNase mixture (On-column PureLink^®^ DNase Treatment, Thermo Fisher Scientific) to remove contaminating DNA. RNA extracted from each sample was eluted in 30 µL of Nuclease-Free Water. RNA concentration (ng/µL) was determined through Qubit^®^ RNA BR Assay Kits (Thermo Fisher Scientifc).

#### 2.4.2. Reverse Transcription (RT) and Real-Time RT-Polymerase Chain Reaction (Real-Time RT-PCR)

A total of 1 µg of RNA was reverse-transcribed by high-capacity cDNA Reverse Transcription Kit (Life Technologies). Samples were incubated in a thermal cycler at 25 °C for 10 min, 37 °C for 2 h, and 85 °C for 5 min.

For all the examined mRNAs, gene expression was determined by quantitative PCR using PowerUpTM SYBRTM Green Master Mix (2×) (Thermo Fisher Scientific). Each amplification reaction was performed in a MicroAmp^®^ Optical 96-well Reaction Plate (Life Technologies, Carlsbad, CA, USA) in a reaction volume of 20 µL made up of 10 µL of SYBR Green, 1 µM of each primer (stock solution 100 µM), 10 ng of cDNA and Nuclease-Free Water. Primer sequences used are reported in Table 1.

The run consisted of 50 °C for 2 min, 95 °C for 2 min, 40 cycles of amplification at 95 °C for 15 s and 60 °C for 1 min in QuantStudio 3 (Thermo Fisher Scientific). QuantStudio™ Design & Analysis Software v1.5.1 (Thermo Fisher Scientific) was used to elaborate gene expression data. The authenticity of the PCR products was verified by melt curve analysis. Each gene expression value was normalized to the expression level of 18S. The fold changes of the investigated genes were expressed in relation to the level of 18S at different time points. The relative abundance of mRNA (relative quantification) was quantified using the comparative ^2−∆∆^Ct method.

### 2.5. Manufacture of Biomaterials Enriched with CAPE Derivatives

Biomaterials were prepared according to the protocol illustrated in Polish Patent no. 235822: Cryogel bone scaffold based on chitosan and bioceramics and the method for its production, and as described in a previous report [45,46,47,48]. In brief, 2% (*w/v*) chitosan (50–190 kDa molecular weight, Sigma-Aldrich Chemicals) and 5% (*w/v*) agarose (gel point 36 ± 1.5 °C, Sigma-Aldrich Chemicals) were suspended in 2% (*v/v*) acetic acid solution (Avantor Performance Materials, Gliwice, Poland) containing appropriate concentrations of the CAPE derivatives (**1a** and **1d**) and mixed. Then, 40% (*w/v*) nanohydroxyapatite (HA) and 2% (*w/v*) sodium bicarbonate (both from Sigma-Aldrich Chemicals) were added and mixed. The obtained paste was subjected to heating (95 °C for 15 min), cooling, and then freezing (−80 °C). The frozen samples were lyophilized (LYO GT2-Basic). Five types of biomaterials were fabricated: with a lower concentration of **1a** (0.5 µM; sample marked as Mat_**1a**_L), with a higher concentration of **1a** (1 µM; sample marked as Mat_**1a**_H), with a lower concentration of **1d** (0.5 µM; sample marked as Mat_**1d**_L), with a higher concentration of **1d** (1 µM; sample marked as Mat_**1d**_H), and a control biomaterial without bioactive compounds (marked as Mat_control). Prior to all experiments, the samples were sterilized by immersion in 70% (*v/v*) ethanol, washed with deionized water for 5 min, and air dried. The microstructure of all fabricated biomaterials (8 mm in diameter and 3 mm in height) was visualized by a stereoscopic microscope (Olympus SZ61TR (Olympus, Tokyo, Japan) scale bar = 1 mm).

### 2.6. Biomaterial Cytotoxicity Assessment

Cytotoxicity assessment of the biomaterials enriched with bioactive compounds was performed through both indirect and direct contact with cells. The cytotoxicity evaluation via indirect contact was determined according to the ISO 10993-5:2009 procedure [45] using 24 h extracts of the biomaterials prepared as it was described earlier [46,47,48,49,50]. First, 1.5 × 10^4^ hFOB 1.19 cells per well were seeded into 96-well plates. After 24 h culture, the medium was replaced with 24 h extracts of the biomaterials (100 mg of biomaterials was incubated in 1 mL of culture medium for 24 h at 37 °C; polystyrene extract served as a negative control of cytotoxicity), and cells were cultured for further 48 h. Then, MTT (Sigma-Aldrich Chemicals) colorimetric assay was conducted to evaluate cell viability as previously described [50]. The results of cytotoxicity analysis were expressed as a percentage of the OD value obtained with the negative control. Cytotoxicity assessment in direct contact with biomaterials was performed by culturing the osteoblasts on the surface of biomaterials. Briefly, biomaterials weighing 50 mg ± 1 mg were placed in 48-well plates and wetted with 150 μL of culture medium. Next, 1.5 × 10^5^ hFOB 1.19 resuspended in 500 μL of medium were seeded onto the surface of the scaffolds. After 48 h of culture, osteoblasts viability was assessed by staining them with a Live/Dead Double Staining Kit (Sigma-Aldrich Chemicals) according to the manufacturer’s procedure. The kit contains calcein-AM and propidium iodide solutions, which stains viable and death cells in green and red, respectively. Stained cells were visualized by a confocal laser scanning microscope (Olympus Fluoview equipped with FV1000, Olympus, Tokyo, Japan).

### 2.7. Pharmacokinetics

#### 2.7.1. Thermal Stability

To ensure the suitability of compounds **1a** and **1d** for adsorption onto biomaterials, their thermal stability was confirmed. Initially, ^1^H-NMR spectra were acquired for the compounds. Subsequently, they were subjected to heating for 20 min at 100 °C by immersing glass tubes containing the solid compounds in a water bath. Following the heating process, their ^1^H-NMR spectra were reacquired and compared with the initial spectra to assess any alterations.

#### 2.7.2. Chemical Stability

For compounds **1a** and **1d**, the chromatographic analyses were performed using a 1260 Infinity II HPLC (Agilent, Santa Clara, CA, USA) consisting of a 1260 Infinity II Quaternary Pump (model G7111A), 1260 Infinity II auto-sampler (model G7129A), a 1260 Infinity II Multicolumn Thermostat (model G7116A), and a 1260 Infinity II Diode Array Detector (model G7115A) equipped with Poroshell 120 EC-C18 (150 × 4.6 mm i.d., particle size 4 µm, Agilent), operating at 20 °C.

Samples were run using a mixture of water and acetonitrile, enriched with trifluoroacetic acid (0.1% *v*/*v*) as mobile phase, in a gradient elution mode starting from 90% to 10% of water over 15 min (Table 2) at a flow rate of 0.8 mL/min. Stock solutions (10 mg/mL) were prepared by dissolving the exact amount of each compound in DMSO and acetonitrile (1:1), and then diluted with acetonitrile to reach 250 µg/mL concentration and analyzed by HPLC, setting the UV-detector at 254 nm.

#### 2.7.3. Kinetic of Chemical Hydrolysis

An amount of 0.02 M of phosphate buffer (PBS, pH 4.5) containing 0.1% Cremophor ELP and 5% DMSO as cosolvents was used to evaluate the chemical stability at pH 4.5, mimicking the osteoclast-mediated degradation acidic microenvironment. CAPE, **1a** and **1d** stock solutions (10 mg/mL) were diluted in prewarmed (37 ± 0.5 °C) PBS solution to reach 10^−4^ M concentration (50 mL); these conditions were used to initiate the reaction. At established time points, samples of 250 µL were withdrawn, filtered, and analyzed by HPLC. The pseudo-first-order rate constant (k*_obs_*) for the compound hydrolysis was calculated from the slops of the linear plots of ln (% residual compound) against time [43,44]. The samples were analyzed in triplicate.

### 2.8. Statistical Analysis

Statistical analysis was performed using Student’s t-test. Results were expressed as mean values ± standard deviation (SD) of at least three replicates. Values of *p* < 0.05 were considered statistically significant and values of *p* < 0.01 were considered highly significant. For data acquired from biomaterials, statistically significant differences among samples were determined by one-way ANOVA followed by Tukey’s test, *p* < 0.05 (GraphPad Prism 8.0.0 Software; GraphPad Software Inc., San Diego, CA, USA).

## 3. Results and Discussions

Stemistry, the use of small molecules to modulate stem cell behaviour, holds promise for tissue repair in RM through ex vivo or in vivo approaches [46,47,48,49]. However, identifying small molecules that effectively enhance regeneration while ensuring bioavailability and stability without inducing toxicity remains a significant challenge, requiring thorough optimization and development efforts [46,47].

### 3.1. Bone Regenerative Ability

DPSCs, extracted from discarded teeth post-extraction, possess remarkable characteristics for regenerative medicine [46,47,48,49,50,51]. Indeed, they exhibit high proliferation rates and ability to differentiate into many cell types, including functional osteoblasts, producing extracellular mineralized matrix and expressing osteogenic markers [50,51,52]. This study addresses the limitations highlighted in previous research by Santos et al. as by focusing on DPSCs, this research aims to determine whether CAPE can modulate the osteoblastic differentiation of stem cell cultures when appropriately stimulated, reinforcing its potential as a molecule for use in bone tissue engineering [53]. Additionally, the compounds tested in this study are derivatives of CAPE with improved pharmacokinetic properties that may enhance its applicability in pharmaceutical preparations [44].

Based on findings from our assays on HGFs [44], compounds **1a**, **1d**, and **2d** at concentrations ranging 0.5–5 µM were chosen to be tested as bone regenerative agents on DPSCs. Compound **2a** was included in the study to verify the importance of the bioisosteres amide with respect to the ester linker and to have a complete comparison of the data, seeking to understand the structure–activity relationship (SAR). Indeed, while compounds **1a** and **2a** contain cinnamate moiety, compounds **1d** and **2d** have caffeate moiety and all of them share the 8-substituted quinoline moiety in their structures (Figure 1).

These molecules demonstrate strong efficacy in promoting cell proliferation and wound closure at low concentrations, making them promising candidates. These results align with the existing literature underscoring the importance of concentration-dependent effects of CAPE in optimizing regenerative outcomes as low concentrations of CAPE effectively stimulate osteogenesis, whereas concentrations above 10 µM reduce cell viability [53,54].

Starting from these premises, we tested their effect on the osteogenic differentiation of DPSCs and their usefulness in the bone biomaterials enriched with the most promising compounds (**1a** and **1d**) was fabricated. Our goal was to propose innovative strategies for the management of bone defects, exploiting the regenerative properties of these CAPE derivatives.

#### 3.1.1. Cell Viability

**Figure 2:**
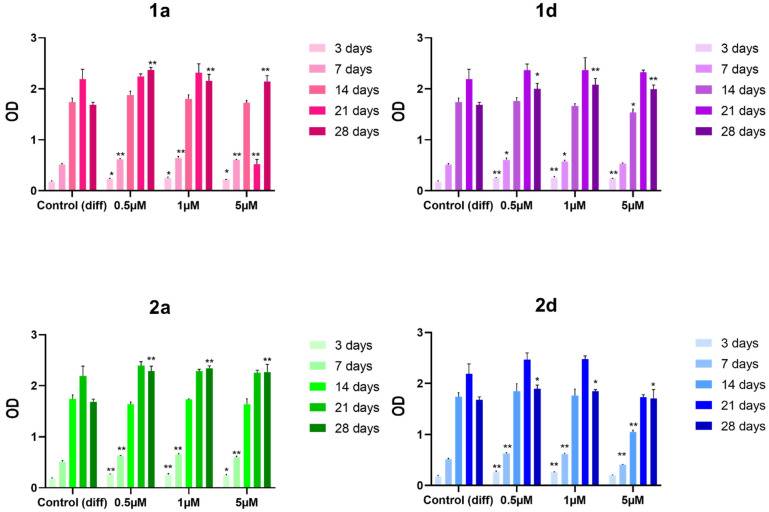
MTT viability test of DPSCs treated with 0.5, 1 and 5 μM concentrations of CAPE derivatives **1a**, **1d**, **2a** and **2d**; * *p* < 0.05; ** *p* < 0.01 vs. its Control (diff). Control (diff): cells in differentiation culture medium; OD: optical density.

First, we tested compounds **1a**, **1d**, **2a** and **2d** for their ability to affect the viability of DPSCs at concentrations of 0.5, 1 and 5 mM and registered the results at five time points (3, 7, 14, 21, and 28 days). Cell viability results indicate that the four derivatives exhibit no toxicity towards DPSCs at all tested concentrations and time points (Figure 2), suggesting promising biocompatibility and supporting the safety and potential applicability of these compounds in regenerative medicine. These findings are supported by previous studies, including those with SAOS-2 osteoblastic cell lines, which indicate that CAPE at similar concentrations does not exhibit cytotoxicity, even though higher concentrations (e.g., 10 µM) show a tendency toward reduced viability [46,47,49,53,54,55]. A related study by Jun et al. also reports toxicity at concentrations above 17.6 µM in long-term cultures of calvarial-derived cells, emphasizing the importance of optimizing CAPE dosages to avoid cytotoxicity [54].

The selected experimental time points allow assessment across early and late differentiation phases, and all compounds show a viability similar to or higher than the differentiation control at all the experimental times, indicating potential dosage stability for long-term application.

The positive effect of CAPE derivatives on DPSC viability corroborates previous findings, including those by Kingsley et al. that demonstrated CAPE’s cytoprotective and growth-promoting properties across DPSC subpopulations and various cell types without disrupting essential cellular functions [55]. CAPE’s role in promoting stem cell survival can be linked to its reported cytoprotective and anti-inflammatory properties, which might mitigate cellular stress during osteogenic differentiation. These findings underscore CAPE’s compatibility with DPSC-based applications, especially potential long-term applications in bone tissue engineering [55]. These results are supported by a study on DPSC grown on 3D scaffold made of caffeic acid (CA)-coated MTA/polycaprolactone (PCL) composites [56].

#### 3.1.2. Extracellular Matrix Deposition Measurement

**Figure 3:**
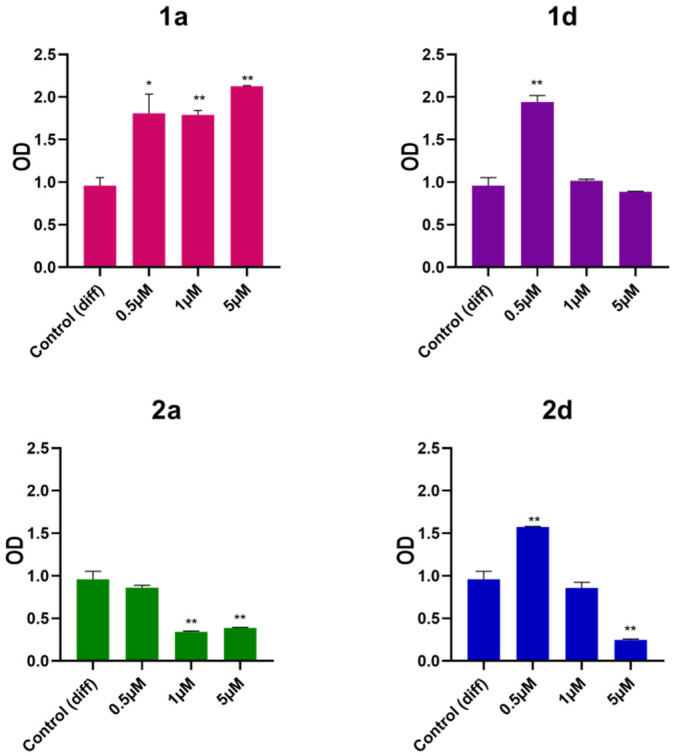
Alizarin red staining of DPSCs induced to differentiate and treated with 0.5, 1 and 5 μM concentrations of CAPE derivatives **1a**, **1d**, **2a** and **2d**. Data shown are the means (±SD) of three independent experiments. * *p* < 0.05; ** *p* < 0.01 vs. Control (diff) 28. Control (diff): cells in differentiation culture medium; OD: optical density.

Extracellular matrix (ECM) calcification marks the final stage of osteogenesis. In this study, Alizarin red staining, an anthraquinone derivative known for its effectiveness in detecting calcium deposits in osteogenic cultures, was employed as a mineralization assay [57,58,59]. After 28 days of exposure to differentiation medium with or without compounds **1a**, **1d**, **2a**, and **2d** at concentrations of 0.5, 1, and 5 µM, extracellular mineralized matrix deposition in DPSCs was assessed by Alizarin Red staining. The findings reveal that compounds **1a**, **1d**, and **2d** are more effective in enhancing matrix deposition compared to non-treated cells, whereas compound **2a** shows no significant changes compared to cells in differentiation medium (Figure 3). Notably, compound **1a** exhibits the most significant increase in matrix deposition across all tested concentrations, whereas compounds **1d** and **2d** only enhance mineralization at the lowest concentration (0.5 µM). As reported in the literature, osteoblastic cells exhibit greater mineralization compared to the control group when exposed to CAPE at concentrations up to 100 nM. However, a reduction in ECM mineralization was observed in the sample exposed to CAPE at 10 μM, highlighting again the concentration-dependent effects of CAPE on optimizing regenerative outcomes [54].

Interestingly, CAPE is effective in inhibiting the pathological calcification of the aortic valve in a model of human aortic valve interstitial cells exposed to an osteogenic medium for 21 days [59]. On the other hand, in physiological conditions, natural phenols, compounds well known for their health benefits [31], are also largely recognized to have a role in improving matrix mineralization during osteogenesis [35,36,38,60,61].

Given these results, compounds **1a**, **1d**, and **2d** were selected for further analysis by using the lower concentration of 0.5 µM, while the effects of compound **2a** were not further investigated.

#### 3.1.3. Alkaline Phosphatase (ALP) Activity

**Figure 4:**
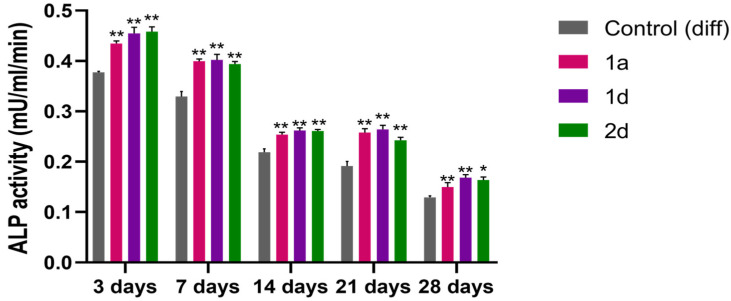
Alkaline phosphatase (ALP) activity in DPSCs induced to differentiate and treated with 0.5 μM concentrations of CAPE derivatives **1a**, **1d** and **2d**. Bar graph shows ALP enzymatic activity (mU/mL/min) after 3, 7, 14, 21, and 28 days of culture; * *p* < 0.05; ** *p* < 0.01 vs. Control (diff) at the same experimental time point. Control (diff): cells in differentiation culture medium.

ALP is a homodimeric protein with phosphorylating properties, which is located in the osteoblastic cell membrane, and plays a key role in hard tissue formation by providing phosphate ions essential for bone ECM mineralization [62]. ALP is highly expressed in mineralized tissue cells and represents an early marker of osteoblastic differentiation [63,64]. To validate Alizarin Red S staining observations and assess mineralization, ALP activity was measured in DPSCs undergoing osteogenic differentiation, treated with compounds **1a**, **1d**, and **2d** at 0.5 µM, and compared to cells exposed to differentiation medium (control (diff)). ALP expression is regulated by complex, interconnected signalling pathways, prompting the selection of 3, 7, 14, 21, and 28-day time points (Figure 4) to capture its activity levels throughout both early and late stages of the process [65]. DPSC cultures exhibit a time-dependent decline in ALP activity, decreasing from 37 × 10^−2^ mU/mL/min at day three to 12 × 10^−2^ mU/mL/min by day 28 (Figure 4). Similarly, DPSCs exposed to compounds **1a**, **1d**, and **2d** exhibit decreasing ALP activity over time, yet significantly higher than non-treated cells at all time points, with slight variations observed among the compounds. Overall, these results suggest that the tested compounds effectively induce osteogenic differentiation in DPSCs.

These findings are consistent with the early production of ALP, commonly found on cell surfaces and in bone and cartilage matrices [66]. During osteogenesis, as certain genes like osteocalcin are upregulated, ALP expression diminishes [65].

These results are in line with previous results showing that CAPE enhances ALP activity in both MG63 cells undergoing osteogenic differentiation and SAOS-2 cultures, which possess a stable osteoblast-like phenotype, following exposure to various CAPE concentrations [67]. Notably, cultures treated with CAPE display extensive mineralized ECM, further supporting CAPE’s role in promoting osteogenic differentiation.

#### 3.1.4. Gene Expression Profile of Mineralization-Related Markers

**Figure 5:**
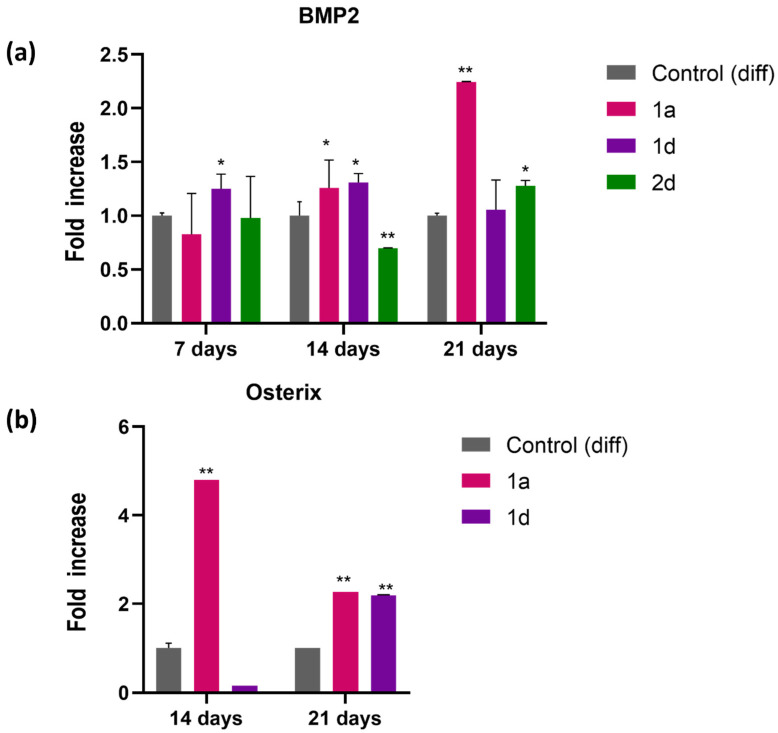
Gene expression of differentiation- and mineralization-associated marker genes in DPSCs induced to differentiate and treated with 0.5 μM concentrations of CAPE derivatives **1a**, **1d** and **2d** (BMP2) and **1a** and **1d** (Sp7). Graphs represent the relative gene expression of (**a**) BMP2 and (**b**) Osterix (SP7) in DPSCs. Data are expressed as fold increase on relative mRNA levels of DPSCs cultivated in growth medium enriched with differentiation agents only. * *p* < 0.05 and ** *p* < 0.01 vs. its Control (diff); Control (diff): cells in differentiation culture medium.

CAPE exerts its multiple biological functions, including osteogenesis, by regulating the expression of various molecules and modulating multiple signalling pathways [31,46,47,49,53]. Previous studies demonstrated that CAPE treatment significantly elevated Bone Morphogenetic Protein 2 (BMP2) expression in a rat model of periodontitis, showing its potential role in enhancing bone formation, with the highest BMP2 expression levels and the most pronounced improvement among the treatment groups [68]. BMP2, a key regulator of osteoblast differentiation and bone formation, initiates early osteogenic processes; its expression increases in the early latency phase and controls the expression of multiple other BMPs, whereas its inhibition can disrupt osteoblast differentiation [69]. BMP2 osteoinductive properties have been exploited to shorten treatment time during distraction osteogenesis, regulating all stages of fracture repair [70,71]. In this study, relative gene expression of BMP2 and SP7 was analyzed using RT-PCR (Figure 5). BMP2 expression was first assessed in DPSCs cultivated in differentiation medium, along with counterparts exposed to 0.5 µM of compounds **1a**, **1d** and **2d**, for 7, 14, 21 days. Notably, BMP2 exhibits varying expression levels among the tested compounds (Figure 5a). Specifically, compound **1d** significantly increased BMP2 expression after 7 days of culture, whereas compound **1a** showed an increase (1.2-fold) after 14 days, further elevated (2.2-fold) by day 21. Conversely, compound **2d** demonstrated higher BMP2 expression only after 21 days of treatment. Given the pivotal role of BMP2 in osteoblast differentiation, further investigation of osteogenic marker expression was focused on compounds **1a** and **1d**. Notably, these compounds are hybrid CAPE derivatives of cinnamic or caffeic acid with a quinoline moiety. Quinoline-containing compounds have been reported to induce bone morphogenetic protein-2 (BMP2) expression in vitro and display osteogenic activity in a murine model in vivo, highlighting their potential for stimulating BMP2-dependent osteogenic differentiation [72]. Gene expression studies further show that caffeic and cinnamic acids can increase the expression of osteoblast-related genes such as bone morphogenetic protein-2 and -7 (BMP2 and BMP7), and osteogenesis-related genes, including ALP, collagen type 1, osterix and osteocalcin (OSC) [73].

Transcription factor Osterix (Osx or Sp7), considered a downstream target of BMP2, is crucial for osteoblast differentiation and is specifically expressed in osteoblasts of all developing bones, as evidenced by the absence of bone formation in Osterix null mice [74]. Once activated, it stimulates the transcription of mature osteoblast genes such as collagen Type I, osteonectin, osteopontin, and bone sialoprotein, all of which are essential for the formation of functional osteoblasts during bone ossification [75,76]. Osx relative gene expression was then analyzed by RT-PCR on DPSCs cultivated in differentiation medium with their counterpart exposed to 0.5 µM of compounds **1a** and **1d** (Figure 5b), for 14 and 21 days. Our findings reveal that the expression of Osx after 14 days shows a notable increase (4.8-fold) in cells exposed to **1a** (Figure 5b). However, after 21 days, there is a consistent enhancement in Osx expression in cells exposed to both compounds, **1a** and **1d** (2.26- and 2.19-fold, respectively, compared to the control sample). Notably, propolis, with CAPE as its major active component, has been shown to enhance runt-related transcription factor 2, Osx, osteocalcin, and type 1 collagen alpha expression, leading to osteoblast differentiation [77].

#### 3.1.5. Collagen Type I Release in DPSC Medium

**Figure 6:**
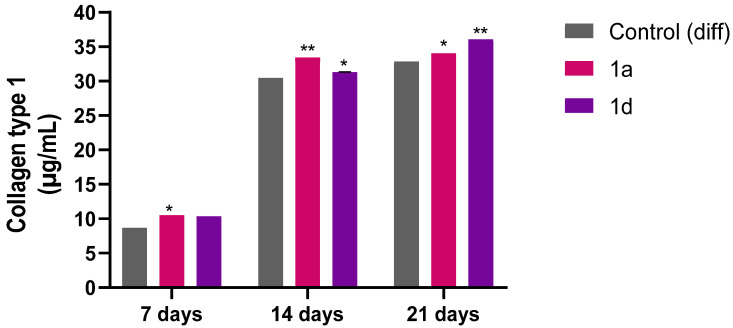
Collagen type I secretion from DPSCs induced to differentiate and treated with 0.5 μM concentrations of CAPE derivatives **1a** and **1d** for 7, 14 and 21 days. Results are normalized on values obtained from crystal violet on the same experiment. * *p* < 0.05 and ** *p* < 0.01 vs. its Control (diff). Control (diff): cells in differentiation culture medium.

To validate the gene expression profile results, the release of Collagen type I was quantified in cell supernatants from cells exposed to compounds **1a** and **1d** at a concentration of 0.5 µM after 7, 14 and 21 days (Figure 6). The results show that the cells exposed to both CAPE derivatives release a higher quantity of Collagen type I with respect to the control sample. These findings align with the literature demonstrating that CAPE-treated injured tissues exhibit enhanced collagen type I deposition, indicating accelerated healing [78].

These results confirm that the combination of two pharmacophoric groups, the caffeic/cinnamic and the quinoline moieties, able to induce osteoinductivity [72,73] has a positive effect on activity, by making a single chemical entity capable of interacting simultaneously on several targets involved in bone regeneration [79] In summary, compounds **1a** and **1d** exhibit a notable capacity to enhance DPSCs differentiation towards an osteogenic lineage, as evidenced by positive outcomes across various osteogenic differentiation assays, including alizarin red staining, ALP activity assessment, and gene expression profiling. These results are in line with the recent literature assessing the role of CAPE, the reference compound for the molecules object of the present study, in inducing osteogenesis [35]. Such findings, together with the antimicrobial properties exerted by our compounds, make compounds **1a** and **1d** multifunctional promising candidates in bone regenerative medicine [43]. Therefore, their potential to enhance the properties of 3D scaffolds for bone regeneration was investigated.

### 3.2. Bone Biomaterials Enriched with Compound ***1a*** and ***1d***

Given the efficacy of the studied compounds in promoting osteogenic differentiation, their potential for enhancing bone biomaterials biocompatibility was also investigated. In this study, biomaterials were fabricated following the protocol described in Polish Patent no. 235822 [80] by using a combination of freeze-drying and gas-foaming techniques to create highly macroporous bone scaffolds composed of a chitosan/agarose matrix reinforced with nanohydroxyapatite. It was previously demonstrated that bone biomaterials fabricated by simultaneous application of freeze-drying and gas-foaming technique allows for obtaining hybrid macroporous biomaterials with open and interconnected porosity which are non-toxic, induce apatite-formation, and are prone to degradation in enzymatic and acidified microenvironments (e.g., osteoclast-mediated) and slow degradation under the physiological pH of 7.4 [48,50] It is worth noting that the open and interconnected porosity of bone implants is crucial for good bone regeneration since it enables cell migration, proliferation, bone ingrowth, implant vascularization, and optimal oxygenation [81,82].

#### Characterization of Fabricated Bone Biomaterials

**Figure 7:**
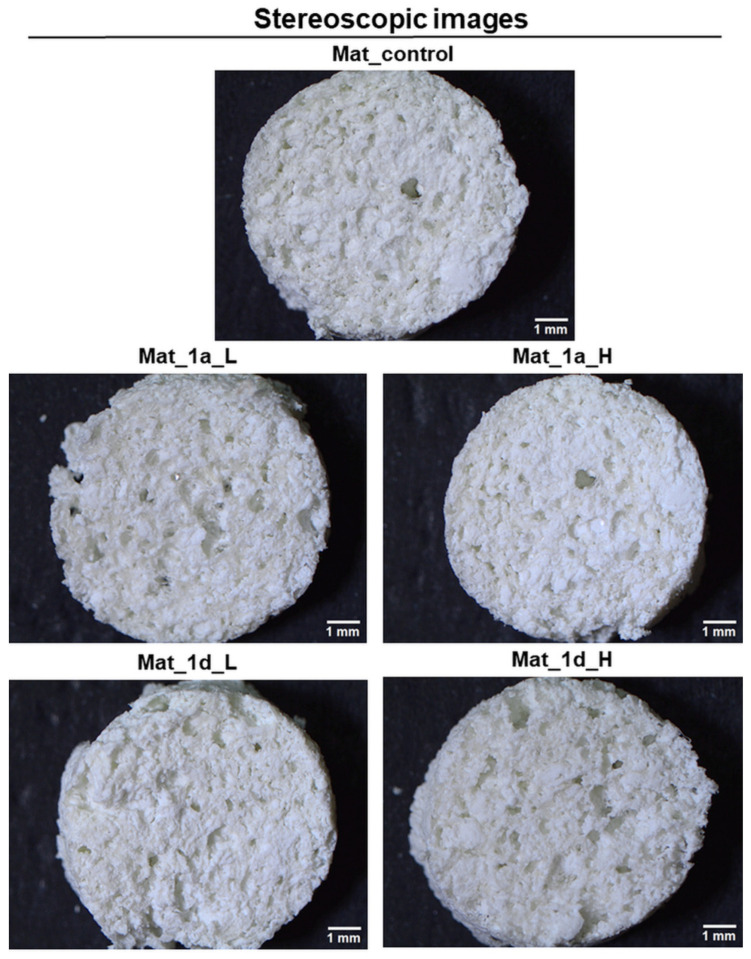
Microstructure of fabricated biomaterials visualized by a stereoscopic microscope (scale bar = 1 mm).

The surface topography of fabricated bone biomaterials was visualized using a stereoscopic microscope (Figure 7). The obtained images show that the biomaterials are characterized by highly macroporous, rough, and ragged microstructure. Microstructural analysis also reveals that there were no apparent changes in the microstructure of the biomaterials upon the addition of CAPE derivatives (mat_**1a** and mat_**1d**) to the base biomaterial (mat_control).

**Figure 8:**
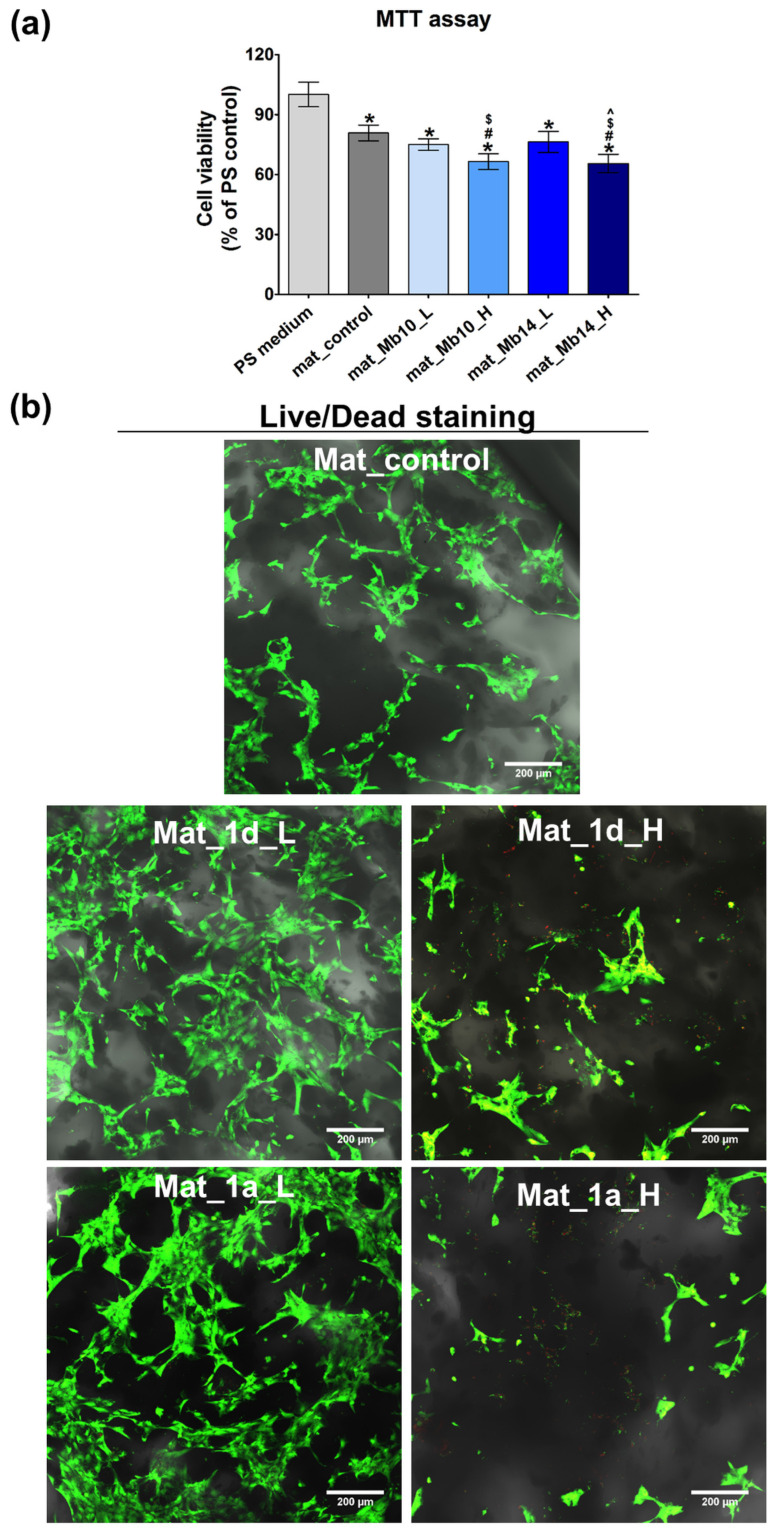
Cytotoxicity assessment of biomaterials enriched with CAPE derivatives on hFOB 1.19 osteoblasts. (**a**) MTT assay conducted by using 24 h extracts of the biomaterials after 48 h cell culture (PS medium—polystyrene extract served as negative control of cytotoxicity; * statistically significant results compared to PS medium, # statistically significant results compared to mat_control, $ statistically significant results compared to mat_**1d**_L, ^ statistically significant results compared to mat_**1a**_L, *p* < 0.05, One-way ANOVA followed by Tukey’s test); (**b**) confocal laser scanning microscope images showing live/dead staining of cells cultured on the surface of biomaterials for 48 h (Nomarski contrast was applied to show biomaterial microstructure; viable cells—green fluorescence; dead cells—red fluorescence; magnified 100×, scale bar = 200 µm).

Cytotoxicity assessment of the fabricated biomaterials enriched with CAPE derivatives was performed through both indirect (Figure 8a) and direct contact with cells (Figure 8b). Two concentrations of the selected two ester compounds **1a** and **1d**, denoted as L for the low content (0.5 µM for **1a** and **1d**) and H for the high content (1 µM for **1a** and **1d**), were chosen to be incorporated into biomaterials microstructure during their production (immobilization by entrapment method). The MTT assay reveals that 24 h conditioned extracts of Mat_control, Mat_**1a**_L, and Mat_**1d**_L decrease the viability of human osteoblasts to 81%, 76%, and 75%, respectively, after 48 h of culture. As per the ISO 10993-5:2009 protocol [45], biomaterials are considered non-toxic if cell viability remains above 70%. Thus, both biomaterials loaded with compounds at the low concentration (Mat_**1a**_L, and Mat_**1d**_L) do not significantly affect hFOB 1.19 cell viability despite the fact that bioceramics-based biomaterials often exhibit ion reactivity, altering ion concentrations in the culture medium, potentially leading to reduced cell viability [83] On the other hand, the same agents incorporated into biomaterials at higher concentration (Mat_**1a**_H and Mat_**1d**_H) decrease cell viability to approx. 65% after 48 h exposure to the extracts, indicating a slight cytotoxicity. The MTT assay results are further supported by fluorescent staining of cells cultured on the surfaces of fabricated biomaterials (Figure 8b). Confocal microscope images reveal a significant number of viable and flattened cells (green fluorescence) on the surfaces of Mat_control, Mat_**1a**_L, and Mat_**1d**_L, confirming non-toxicity and good cell attachment. Conversely, surfaces of Mat_**1a**_H and Mat_**1d**_H appear sparsely populated by viable cells (green fluorescence) but contain numerous dead cells (red fluorescence).

In the literature, there are few examples of biomaterials coated with caffeic acid in order to improve biocompatibility and osteogenic properties [84,85,86] Although they are promising, further carefully designed studies are necessary to demonstrate the relationship between the potential of caffeic acid derivatives and RM. Nonetheless, some issues need to be overcome before proceeding further. Despite the many beneficial properties of CA and its derivatives, as also demonstrated in the present paper, these compounds have a very low stability. To produce more stable compounds than the reference one with the same or improved osteogenic properties, the chemical and thermal stability of compounds **1a** and **1d** was investigated, to confirm the accuracy of our strategy of synthesis.

### 3.3. Chemical and Thermal Stability

#### 3.3.1. Thermal Stability

**Figure 9:**
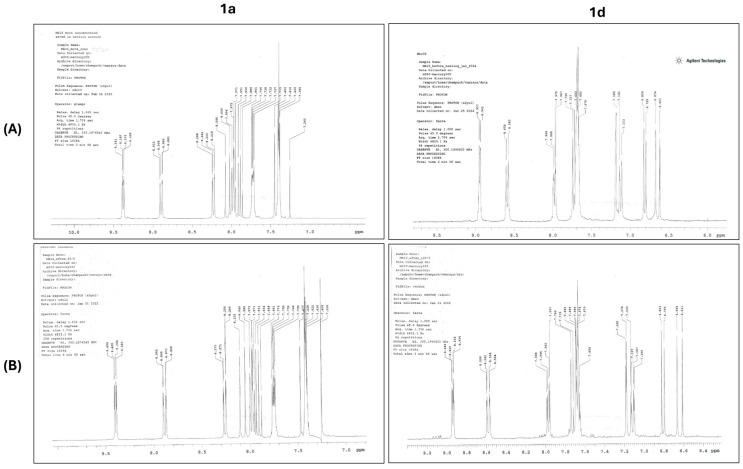
Main signals of ^1^H NMR of compounds **1a** and **1d** (**A**) after and (**B**) before the heating.

Studies highlighted that CAPE’s chemical stability is significantly influenced by temperature, posing limitations on its application in pharmaceutical fields [87] CAPE decomposition was observed in plasma samples at room temperature, with increased degradation at 37 °C and slowed degradation at 4 °C [87] In our previous study [44], results demonstrated that CAPE derivatives **1a** and **1d** remain stable for 30 min at 50 °C, as evidenced by consistent ^1^H NMR spectra before and after exposure to heat. Thermal stability may be attributed to the presence of 8-quinoline which, through its steric hindrance and spatial geometry, protects the ester function from hydrolysis. Since an immersion step of 15 min in a water bath kept at a temperature of 95 °C was a necessary step of the process of biomaterials fabrication enriched with compounds **1a** and **1d**, heat stability of compounds **1a** and **1d** was measured for 20 min in a water bath kept at a temperature of 100 °C [44]. The results show that both compounds remain stable for 20 min at this temperature, as indicated by their identical ^1^H NMR spectra before and after exposure to heat (Figure 9), suggesting the suitability of the process for the fabrication of these biomaterials.

#### 3.3.2. Chemical Stability

Bone remodelling, essential for repairing bone tissue, hinges on the harmonized interplay between osteoclasts and osteoblasts to achieve coordination between bone formation and resorption [88] Osteoclasts play a pivotal role in the latter by producing hydrochloric acid, which dissolves the bone mineral, facilitating the remodelling process [88] Conversely, osteoblasts, responsible for new bone deposition, function optimally at a pH of 7.4. In more acidic environments, such as those created during bone resorption, osteoblasts are unable to mineralize the bone collagen matrix effectively. Interstitial tissue pH typically ranges between 7.0 and 7.2, emphasizing the importance of pH balance in bone metabolism [88,89]. The chemical stability of the best compounds, **1a** and **1d**, was evaluated in 0.02 M phosphate buffer solutions at pH 4.5 and 7.4, which represent the pH of the acidic microenvironment of osteoclast-mediated degradation and the pH at which osteoblasts function optimally, respectively. Results were compared with that obtained for the reference compound CAPE, whose stability decreases with the increase in the pH, having t_1/2_ of 38.5 h at pH 7.4 and of 117 h at pH 4.5 (Table 3).

On the other hand, **1a** was the most stable molecule when pH arises and shows no degradation at pH 7.4 while having t_1/2_ of 80.6 h at pH 4.5, whereas **1d** results in less stability when compared to **1a** having t_1/2_ of 21.19 h at pH 7.4 while showing no degradation at pH 4.5. Such results are in agreement with what is already reported for phenols [90]. In conclusion, these findings suggest that the CAPE derivatives **1a** and **1d** are chemically stable compounds in both acidic and physiological pH. Their chemical and thermal stability, coupled with their bone regenerative potential, makes them promising candidates for use in fabricating bone biomaterials for applications in regenerative medicine.

Further studies should be aimed towards in vivo validation of the promising in vitro results obtained in this study, to move towards a clinical translation.

## 4. Conclusions

**Figure 10:**
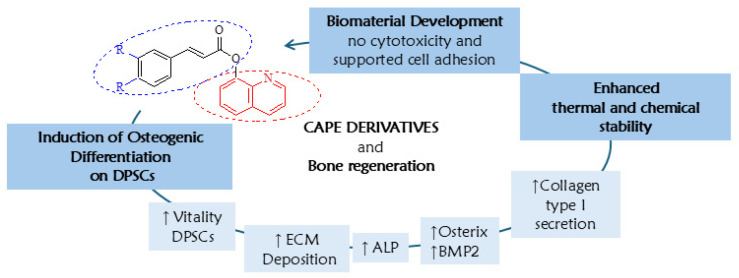
Summary of the main findings of this paper.

As shown in Figure 10, the study demonstrated the potential of CAPE derivatives **1a** and **1d** in enhancing DPSCs’ osteogenic differentiation, as evidenced by various assays including cell viability, extracellular matrix deposition, alkaline phosphatase activity, and gene expression profiling of key osteogenic markers. These two compounds exhibited no cytotoxicity and significantly enhanced mineralization-related markers, suggesting a promising role in bone regeneration. The thermal and chemical stability of these compounds in different conditions positions them as promising candidates for bone biomaterial fabrication. Indeed, manufacture of bone biomaterials enriched with compounds **1a** and **1d** was biocompatible at low concentrations, further supporting their suitability for use in regenerative medicine applications. Overall, this study proposes innovative strategies for the management of bone defects by leveraging the regenerative properties of CAPE derivatives, paving the way for future advancements in bone regenerative therapy.

## Figures and Tables

**Table 1:** Primer sequences for quantitative PCR.

Gene	Sequence (5′ to 3′)
18S_FOR	CATGGCCGTTCTTAGTTGGT
18S_REV	CGCTGAGCCAGTCAGTGTAG
BMP2_FOR	CACTTGGCTGGGGACTTCTT
BMP2_REV	CGCGCAGTCTCTCTTTTCAC
SP7_FOR	CTCAGGCCACCCGTTG
SP7_REV	CATAGTGAACTTCCTCCTCAAGC

**Table 2:** Chromatographic conditions.

Time (min)	A (%)	B (%)
0	90	10
6	10	90
10	10	90
12	90	10
15	90	10

**Table 3:** Chemical stability of CAPE, and compounds **1a** and **1d**.

CAPE			1a		1d	
	t_1/2_ (h) ^a^	K_obs_ (h^−1^) ^a^	t_1/2_ (h) ^a^	K_obs_ (h^−1^) ^a^	t_1/2_ (h) ^a^	K_obs_ (h^−1^) ^a^
pH 4.5	117.5 (±2.10)	0.006 (±0.0009)	80.6 (±2.3)	0.009 (±0.0008)	stable	-
pH 7.4	38.5 (±0.41)	0.018 (±0.004)	stable	-	21.19 (±0.37)	0.033 (±0.002)

^a^ Values are means of three experiments.

## Data Availability

The raw data supporting the conclusions of this article will be made available by the authors on request.

## Abbreviations

The following abbreviations are used in this manuscript:

ALP	Alkaline Phosphatase activity
BMP	Bone Morphogenetic Protein
CA	Caffeic Acid
CAPE	Caffeic Acid Phenethyl Ester
CFU-O	Colony-Forming Unit-Osteoprogenitor
DPSCs	Dental Pulp Stem Cells
ECM	Extracellular Matrix
FBS	Fetal Bovine Serum
hFOB	Human Fetal Osteoblast Cell Line
OSC	Osteocalcin
Osx	Osterix
PB	Phosphate Buffer
PCL	Polycaprolactone
RM	Regenerative Medicine
SD	Standard Deviation
SAR	Structure–Activity Relationship
